# Supplementary material for: Targeted modulation of protein liquid–liquid phase separation by evolution of amino-acid sequence
Source: PLoS Comput Biol. 2021 Aug 24;17(8):e1009328. doi: 10.1371/journal.pcbi.1009328 (PMC8415608; doi:10.1371/journal.pcbi.1009328)
Supplement: S1 Text — Includes Table A. Table A: Table of amino acids. The 20 naturally occurring amino acids with their one- and three-letter codes, alongside their charges. Amino acids marked with a ‘⋆’ are aromatic. The last columns give the λ- and σ-parameters which define the hydrophobicity scale. (PDF) [file pcbi.1009328.s001.pdf]

Supplementary material:

Targeted modulation of protein liquid–liquid phase separation by evolution of amino-acid sequence

Simon M. Lichtinger,[1, a)] Adiran Garaizar,[2] Rosana Collepardo-Guevara,[1, 2, 3] and Aleks Reinhardt[1]

[1)] *Yusuf Hamied Department of Chemistry, University of Cambridge, Lensfield Road, Cambridge, CB2 1EW, United Kingdom*

[2)] *Department of Physics, University of Cambridge, Cavendish Laboratory, Maxwell Centre, JJ Thomson Avenue, Cambridge, CB3 0HE, United Kingdom*

[3)] *Department of Genetics, University of Cambridge, 20 Downing Place, Cambridge, CB2 3EJ, United Kingdom*

(Dated: 8 August 2021)

S1 COMPUTATIONAL DETAILS

S1-a Coarse-grained model of proteins

The coarse-grained potential of proteins introduced by Dignon and co-workers[1] is based on an amino-acid level description of protein chains. Each amino acid is represented by a bead. For amino acids that are covalently bonded in the protein of interest, their beads are connected by a harmonic spring,

$$\Phi_{\rm harm} = \frac{1}{2}k(r - r_0)^2, \tag{S1}$$

where $k = 19.2\,\mathrm{kcal\,mol^{-1}\,Å^{-2}}$ is the spring constant and $r_0 = 3.81$ Å is the equilibrium bond length. Beads interact with one another through an Ashbaugh–Hatch modulated[2] hydrophobicity-scaled (12,6)-Lennard-Jones (LJ) potential,

$$\Phi(r)_{ij} = \begin{cases} \Phi_{\rm LJ}(r) + (1 - \lambda_{ij})\varepsilon_{ij} & \text{if } r < 2^{1/6}\sigma_{ij}, \\ \lambda_{ij}\Phi_{\rm LJ}(r) & \text{otherwise,} \end{cases} \tag{S2}$$

TABLE A. **Table of amino acids.** The 20 naturally occurring amino acids with their one- and three-letter codes, alongside their charges. Amino acids marked with a ‘⋆’ are aromatic. The last columns give the λ- and σ-parameters which define the hydrophobicity scale.

Full name	Code		Charge	λ	σ/Å
Arginine	Arg	R	+	0.0	6.56
Aspartate	Asp	D	−	0.378	5.58
Asparagine	Asn	N	0	0.432	5.68
Glutamate	Glu	E	−	0.459	5.92
Lysine	Lys	K	+	0.514	6.36
Histidine	His	H	+	0.514	6.08
Glutamine	Gln	Q	0	0.514	6.02
Cysteine	Cys	C	0	0.595	5.48
Serine	Ser	S	0	0.595	5.18
Glycine	Gly	G	0	0.649	4.50
Threonine	Thr	T	0	0.676	5.62
Alanine	Ala	A	0	0.730	5.04
Methionine	Met	M	0	0.838	6.18
⋆ Tyrosine	Tyr	Y	0	0.865	6.46
Valine	Val	V	0	0.891	5.86
⋆ Tryptophan	Trp	W	0	0.946	6.78
Leucine	Leu	L	0	0.973	6.18
Isoleucine	Ile	I	0	0.973	6.18
Proline	Pro	P	0	1.0	5.56
⋆ Phenylalanine	Phe	F	0	1.0	6.36

where r is the interparticle distance, and

$$\Phi_{\rm LJ}(r) = 4\varepsilon_{ij}\left[\left(\frac{\sigma_{ij}}{r}\right)^{12} - \left(\frac{\sigma_{ij}}{r}\right)^{6}\right], \tag{S3}$$

where ε_{ij} is the minimum of the LJ potential, σ_{ij} is the LJ diameter and λ_{ij} is a hydrophobicity scaling parameter. In each case, i and j correspond to the amino acid types of the two particles. Residues that carry a charge (see Table A) also interact with a Debye-screened[3] electrostatic potential,

$$\Phi_{\rm coul}(r) = \frac{q_i q_j}{4\pi D r}\mathrm{e}^{-r/\kappa}, \tag{S4}$$

where D is the permittivity $D = 80\,\varepsilon_0$ (where ε_0 is the electric constant), κ is the screening length ($\kappa = 1$ nm, corresponding to an ionic strength of 100 mM) and q_i and q_j are the charges of the amino acids.

In the work of Dignon and co-workers,[1] two possibilities for assigning λ_{ij}, ε_{ij} and σ_{ij} are presented. Here, we use the variant based on the Kim–Hummer (KH) model,[4] which has been brought into the form of equation (S2) with parameters

$$\varepsilon_{ij} = |\alpha(\varepsilon_{\rm MJ} - \varepsilon_0)| \quad \text{and} \quad \lambda_{ij} = \begin{cases} 1 & \text{if } \varepsilon_{\rm MJ} \le \varepsilon_0, \\ -1 & \text{otherwise,} \end{cases} \tag{S5}$$

where $\varepsilon_{\rm MJ}$ is the Miyazawa–Jerningan empirical contact potential,[5] and $\alpha = 0.228$ and $\varepsilon_0 = 1\,\mathrm{kcal\,mol^{-1}}$ are benchmarked on experimental radii of gyration. The σ_{ij} parameters are arithmetic means of effective Van der Waals radii of the amino acids, $\sigma_{ij} = (\sigma_i + \sigma_j)/2$, which are given in Table A.

A version of this model with a new parameter set[6] was recently introduced, featuring a temperature dependence of the λ_{ij} parameters to match experimental data more closely. We have not employed this potential here, as we are primarily interested in relative shifts of $T_{\rm c}$ and not its absolute value.

As an alternative to the Kim–Hummer model, varying $\lambda_{ij} = (\lambda_i + \lambda_j)/2$ parameters in an amino-acid specific way is also possible, while taking ε_{ij} to be constant. This has been shown to yield comparable results.[1] Whilst we do not employ this model here, we use these λ_i values (Table A) of the amino acids to quantify their hydrophobicity.

S1-b Determining phase coexistence: simulation details

We studied LLPS with direct-coexistence molecular dynamics simulations[7] in which a high- and a low-density phase coexist. Molecules can be exchanged between the two phases, allowing the densities to equilibrate across the interface [Fig. S1(A))]. In the limit of sufficiently large systems, where the interface becomes negligible compared to the bulk of each phase, this approach allows us to determine the densities of the two compositional phases. Direct-coexistence simulations provide an

[a)] Present address: Department of Biochemistry, University of Oxford, 3 South Parks Road, Oxford, OX1 3QU, United Kingdom

especially simple method of determining phase equilibria, particularly with liquid-like phases considered here. Since phase separation above the spinodal is a nucleation-initiated process, hysteresis may be a problem, and direct-coexistence simulations may require a careful calibration of the interface at the start of a simulation.[8] However, with the kinds of coarse-grained potentials we are using, phase transitions are facile and an interface forms readily. Direct-coexistence simulations have therefore routinely been employed in computational studies of LLPS with such models.[9]

In order to construct a phase diagram, we perform direct-coexistence simulations at a number of temperatures [Fig. S1(A)]. We determine the densities of the coexisting phases by binning particles along the z axis and finding a least-squares best fit to two constant densities – with an interface of a system-specific width – to identify the low- and the high-density phases as well as the interfacial region [Fig. S1(B))]. Finally, the densities we extract are plotted on a temperature–density phase diagram, as for example in Fig. S2. To interpolate the data points close to the critical temperature, we use an empirical fit[10] which, although it does not capture the behaviour of the system very well at low temperatures, is sufficient to find the approximate critical temperature and density. In particular, we fit simultaneously to

$$(\rho_{\mathrm{high}}(T) - \rho_{\mathrm{low}}(T))^{3.06} = d\,(1 - T/T_{\mathrm{c}}) \qquad \text{and} \qquad \text{(S6)}$$

$$\rho_{\mathrm{high}}(T) + \rho_{\mathrm{low}}(T) = 2\rho_{\mathrm{c}} + 2s_2(T_{\mathrm{c}} - T), \qquad \text{(S7)}$$

where $\rho_{\mathrm{high}}(T)$, $\rho_{\mathrm{low}}(T)$ and ρ_{c} are the densities of the high-density and low-density phases and the critical density, respectively; T_{c} is the critical temperature and d and s_2 are fitting parameters. This works by numerically finding a best fit for the four constants – ρ_{c}, T_{c}, d and s_2 – from equations constrained by all pairs of $\rho_{\mathrm{high}}(T)$ and $\rho_{\mathrm{low}}(T)$ deemed by inspection of the curvature of the observed data series to lie below T_{c}.

We note that in direct-coexistence simulations, above the critical temperature, the system forms a single supercritical fluid; the density fitting outlined above, which assumes two distinct phases have formed, will thus produce non-physical results reflecting the natural density fluctuations of the system; any densities so determined do not correspond to coexisting phases. Nevertheless, such spurious points help us to ascertain that we have already crossed the critical point, forming a characteristic ‘protrusion’ of the coexistence region towards higher temperatures. The presence of such features at the appropriate point can thus provide an additional check that we have determined the critical temperature correctly, and so we have included them in phase diagrams as greyed-out points for reference.

We performed molecular dynamics simulations with the Lammps simulation package,[11] using a velocity Verlet integrator with a time step of $\delta t = 10\,\mathrm{fs}$. Direct-coexistence simulations were run in the canonical ensemble with a Langevin thermostat[12] with a damping time of $10^4\,\delta t$. We used a tetragonal simulation box with periodic boundary conditions, with typical dimensions 134.4 Å × 134.4 Å × 403.2 Å with 64 chains for FUS and hnRNPA1, and 267.2 Å × 267.2 Å × 1336.2 Å with 512 chains for LAF1. We have verified that for typical systems, these numbers of copies of the polymer chain were sufficient to ensure that finite-size effects did not dominate the system's bulk behaviour; to this end, we simulated systems ~30 % smaller and verified that the mean densities computed for these smaller systems were sufficiently similar that the estimated critical temperature fell within 5 K of the original one, which is within typical error bars of temperature measurements with Langevin thermostats.

S2 HYDROPHOBIC SEQUENCE OPTIMUM

Figure S2 shows the phase diagram of a possible optimum of hydrophobicity-driven LLPS within the CG model. While assessing an optimum of the charge-driven case is difficult, it possible to make a guess about the scaled LJ potential by inspection of the model parameters. The highest ε_{ij} value is for a Phe–Phe interaction, which is also longer-ranged than for most other amino acids. The phase diagram shown illustrates how large the changes in critical temperature can be, but it also demonstrates that trivially optimising the critical temperature results in a biologically uninteresting sequence.

S3 DUMMY GENETIC ALGORITHM

Figure S3 shows the genetic algorithm progression for FUS with enhanced phase separation, contrasted with a dummy genetic algorithm without any selection pressure. By setting the tournament size $N_{\mathrm{tour}} = 1$, we remove any favouring of fit parents for the generation of children, and instead of performing weak-population replacement, we replace a random individual within the population with an acceptance probability of 0.5. However, we leave all mutagenesis steps intact, thus setting up a control for how the random mutagenesis itself affects FUS phase separation. In Fig. S3, we show that such a dummy genetic algortihm exhibits very little evolutionary driving force compared to our original implementation. The observed increases in phase-separating ability are therefore not due to mere random alterations of the sequences, but are directly driven by our genetic algorithm.

S4 EVOLUTION WITH THE CATION–π MODEL

In order to determine how sensitive the results of the predictions of the genetic-algorithm runs are to the choice of model, we have re-run evolution simulations of FUS and hnRNPA1 with a reparameterised coarse-grained potential of Das *et al.*,[13] which we refer to as the cation–π model. The approach is similar to the KH model we introduced in Section S1-a; each amino acid in the sequence is represented by a bead and the primary interaction is still of a Lennard-Jones type coupled with Debye–Hückel ionic terms, but the cation–π model uses the hydrophobicity scale directly, i.e. by changing λ rather than ε values, and additionally accounts for cation–π interactions for arginine and leucine with phenylalanine, tryptophan and tyrosine residues with an additional Lennard-Jones interaction. We use ‘scheme (i)’ from the paper of Das *et al.*[13] in our implementation, and identical parameters for the harmonic spring bond length and spring constant.

We show the results of the evolution of FUS and hnRNPA1 with our genetic algorithm in Fig. S6. These results are largely comparable with the results shown in Fig. 1(B) of the main text for FUS and Fig. 4(A) of the main text for hnRNPA1. Although the change in the fitness function as a function of the genetic-algorithm run is slower than that for the KH-model analogue, this is not unexpected, since the width of the phase diagram, which is how the fitness function is determined, varies less with the critical temperature in the cation–π model [as we show in Fig. 7 of the main text]. While some model-specific differences are expected, we can see from Fig. S6(B) that these amount mainly to a small increase in favourability of cations, which is expected from the goal of the reparameterisation of the cation–π potential, and a slight narrowing of the broadness of

the hydrophobic distribution of residues generated. Although of course these models are ultimately based on the same coarse-graining philosophy, and so the good agreement between their predictions is perhaps not entirely unexpected, these results suggest that the broad trends that we have discussed in the main text are not overly sensitive with respect to the choice of model.

S5 LIQUID CHARACTER OF PHASES

When studying LLPS, it is important to control the liquid character of the observed phases, as proteins can also undergo gelation[14] and glass transitions.[15] While the study of general polymer dynamics in the CG model is not the objective of this work, and dynamical properties of coarse-grained models do not usually faithfully reproduce real dynamics, we nevertheless computed the per-bead diffusion coefficient for our systems to ascertain that a glass transition has not occurred. Ballistic and sub-diffusive regimes may precede the diffusive regime, and in polymer systems, several sub-diffusive regimes can often be observed.[16,17] The diffusion coefficient D can be obtained by the Einstein relation[18] for the mean squared displacement, $\langle \Delta r^2 \rangle = 6Dt$, which holds in the diffusive regime of *long* times t. We have computed diffusion coefficients in this way over a range of temperatures, and show these alongside the phase diagram of hnRNPA1-WT and one of the evolved sequences in Fig. S7. In all cases, the diffusive regime can easily be reached in a brute-force simulation at readily accessible time scales, suggesting that the systems are not dynamically arrested under the conditions of interest. The variation in the diffusion coefficients is almost entirely due to the density; the temperature dependence simply corresponds to the fact that the liquid phase is less dense at a given temperature for the wild-type than it is for the evolved sequence. The diffusion coefficients shown are only qualitative, in the sense that in the potential we use, many degrees of freedom have been coarse-grained away, and the unit of time is not directly comparable to experiment. However, ratios of diffusion coefficients are nevertheless meaningful. The temperature dependence of the diffusion coefficients is non-linear; however, there is no obvious discontinuous change in the diffusion coefficient as a function of temperature in these data even at low temperatures in the liquid phase, which we take as justification that our simulations describe LLPS rather than glass formation, which could complicate our interpretation of the results.

S6 AMINO-ACID SEQUENCES OF PROTEINS STUDIED

We give below the amino-acid sequences of the prion-like IDR of FUS, hnRNPA1-IDR and LAF1-IDR[19] studied in this work, using one-letter codes [Table A] for the amino acids.

[residues 1–163 of UniProt sequence P35637-1]

FUS MASND YTQQA TQSYG AYPTQ PGQGY SQQSS QPYGQ
QSYSG YSQST DTSGY GQSSY SSYGQ SQNTG YGTQS
TPQGY GSTGG YGSSQ SSQSS YGQQS SYPGY GQQPA
PSSTS GSYGS SSQSS SYGQP QSGSY SQQPS YGGQQ
QSYGQ QQSYN PPQGY GQQNQ YNS

[residues 186–320 of UniProt sequence P09651-2]

hnRNPA1 MASAS SSQRG RSGSG NFGGG RGGGF GGNDN FGRGG
NFSGR GGFGG SRGGG GYGGS GDGYN GFGND GSNFG
GGGSY NDFGN YNNQS SNFGP MKGGN FGGRS SGPYG
GGGQY FAKPR NQGGY GGSSS SSSYG SGRRF

[residues 2–168 of UniProt sequence D0PV95-1]

LAF1 ESNQS NNGGS GNAAL NRGGR YVPPH LRGGD GGAAA
AASAG GDDRR GGAGG GGYRR GGGNS GGGGG GGYDR
GYNDN RDDRD NRGGS GGYGR DRNYE DRGYN GGGGG
GGNRG YNNNR GGGGG GYNRQ DRGDG GSSNF SRGGY
NNRDE GSDNR GSGRS YNNDR RDNGG DG

S7 EVOLVED SEQUENCES

Below, we provide example output sequences, taken from the final populations of the appropriate GA runs as described in this work, using one-letter codes [Table A]. The residues that have changed compared to the initial sequence are highlighted in red. Sequences of full populations as a function of the progression of all our GA runs can be found in the supporting data. In the list below, for the case of FUS, 'max' and 'min' refer to the sequences with the highest and lowest critical temperatures at the end of the genetic-algorithm run when the fitness function was designed to increase and to decrease the width of the phase diagram, respectively.

FUS max MASFD YLMYA QQSYG AYGTQ PYQIY SQQSP QPYHM
QPYSG YSQST YTSGY GMSSM YPYGQ SQNTG YGTQS
WPLGY GSTGG CGSSQ SSQSS IGQQG SYWGY GQQPA
PSSTS YFYGS SSQSS SWGQK QSGSY SQLPS YGGQQ
YSYGQ QQSYN PHQGY WQQWQ YHS

FUS min MASNM YPQQA TQSYG AYRTQ PGTGY SKQSS QPYGQ
QSYKG YCGVT GTSGE GQSSY KSYGQ SQNTG SGTQS
KPQGY GSTGG YGSSQ GSKSK PGQQS SYNGI GQQPA
RSSTS GSYGG KSQSS SYGQP QSGSP SQQPS DGGQQ
QSGGQ QQSYN PPQGY GQQQQ YND

hnRNPA1 FASAS SSQRG NSGSG NFPGC TIDGF GKNDN FGNGG
NFSGR GWFGG CRGGP WYGFS GDGYN GFGND GSNFG
YFVSY NDFGN YNEQS SNFDP MRNGN FIGYS SGPYG
GGGQF FARFR IQGGY GGSSS SSSYM SGRRF

LAF1 ESNQS NNGGH GYAAL NRGGR YVPPH LRGGD GGAAA
AASAG GDDRR GGAGG GFYRR GGGNS GNGGG GDYDR
GYNDN RDDRD NRGGS GGYGW PRNYE DRGYN GGGGA
GGNRS YNNNR GGGEV GYNRQ DRGDG GSSNF SRGDY
NNRDE GSDNR GSGRS YNNDR RDNGG DG

S8 SEQUENCES USED IN EXPERIMENTAL VALIDATION

For benchmarking the predictions of the models used, we have considered the following sequences of hnRNPA1 IDP variants, using the nomenclature of Bremer and co-workers.[20] The names of the variants correspond to the one-letter amino-acid codes of residues that replace those in the wild-type; they are highlighted in red below.

WT MASAS SSQRG RSGSG NFGGG RGGGF GGNDN FGRGG
NFSGR GGFGG SRGGG GYGGS GDGYN GFGND GSNFG
GGGSY NDFGN YNNQS SNFGP MKGGN FGGRS SGPYG
GGGQY FAKPR NQGGY GGSSS SSSYG SGRRF

−3R+3K MASAS SSQRG KSGSG NFGGG RGGGF GGNDN FGRGG
NFSGR GGFGG SKGGG GYGGS GDGYN GFGND GSNFG
GGGSY NDFGN YNNQS SNFGP MKGGN FGGRS SGGSG
GGGQY FAKPR NQGGY GGSSS SSSYG SGRKF

−4F−2Y MASAS SSQRG RSGSG NSGGG RGGGF GGNDN FGRGG

	NSSGR GGFGG SRGGG GYGGS GDGYN GFGND GSNSG<br>GGGSS NDFGN YNNQS SNFGP MKGGN FGGRS SGGSG<br>GGGQY SAKPR NQGGY GGSSS SSSSG SGRRF
**−6R+6K**	MASAS SSQKG KSGSG NFGGG RGGGF GGNDN FGKGG<br>NFSGR GGFGG SKGGG GYGGS GDGYN GFGND GSNFG<br>GGGSY NDFGN YNNQS SNFGP MKGGN FGGKS SGGSG<br>GGGQY FAKPR NQGGY GGSSS SSSYG SGRKF
**+7F−7Y**	MASAS SSQRG RSGSG NFGGG RGGGF GGNDN FGRGG<br>NFSGR GGFGG SRGGG GFGGS GDGFN GFGND GSNFG<br>GGGSF NDFGN FNNQS SNFGP MKGGN FGGRS SGGSG<br>GGGQF FAKPR NQGGF GGSSS SSSFG SGRRF
**+7K+12D**	MASAD SSQRD RDDKG NFGDG RGGGF GGNDN FGRGG<br>NFSDR GGFGG SRGDG KYGGD GDKYN GFGND GKNFG<br>GGGSY NDFGN YNNQS SNFDP MKGGN FKDRS SGPYD<br>KGGQY FAKPR NQGGY GGSSS SKSYG SDRRF
**+7R**	MASAS SSQRG RSGRG NFGGG RGGGF GGNDN FGRGG<br>NFSGR GGFGG SRGGG RYGGS GDRYN GFGND GRNFG<br>GGGSY NDFGN YNNQS SNFGP MKGGN FRGRS SGPYG<br>RGGQY FAKPR NQGGY GGSSS SRSYG SGRRF
**+7R+12D**	MASAD SSQRD RDDRG NFGDG RGGGF GGNDN FGRGG<br>NFSDR GGFGG SRGDG RYGGD GDRYN GFGND GRNFG<br>GGGSY NDFGN YNNQS SNFDP MKGGN FRDRS SGPYD<br>RGGQY FAKPR NQGGY GGSSS SRSYG SDRRF
**−9F+3Y**	MASAS SSQRG RSGSG NFGGG RGGGY GGNDN GGRGG<br>NYSGR GGFGG SRGGG GYGGS GDGYN GGGND GSNYG<br>GGGSY NDSGN GNNQS SNFGP MKGGN YGGRS SGGSG<br>GGGQY GAKPR NQGGY GGSSS SSSYG SGRRS
**−12F+12Y**	MASAS SSQRG RSGSG NYGGG RGGGY GGNDN YGRGG<br>NYSGR GGYGG SRGGG GYGGS GDGYN GYGND GSNYG<br>GGGSY NDYGN YNNQS SNYGP MKGGN YGGRS SGGSG<br>GGGQY YAKPR NQGGY GGSSS SSSYG SGRRY

S9 REFERENCES

[1] G. L. Dignon, W. Zheng, Y. C. Kim, R. B. Best, and J. Mittal, "Sequence determinants of protein phase behavior from a coarse-grained model," PLoS Comput. Biol. **14**, 1 (2018).

[2] H. S. Ashbaugh and H. W. Hatch, "Natively unfolded protein stability as a coil-to-globule transition in charge/hydropathy space," J. Am. Chem. Soc. **130**, 9536 (2008).

[3] P. Debye and E. Hückel, "Zur Theorie der Elektrolyte. I. Gefrierpunkt-serniedrigung und verwandte Erscheinungen," Phys. Z. **24**, 185 (1923).

[4] Y. C. Kim and G. Hummer, "Coarse-grained models for simulations of multiprotein complexes: Application to ubiquitin binding," J. Mol. Biol. **375**, 1416 (2008).

[5] S. Miyazawa and R. L. Jernigan, "Residue–residue potentials with a favorable contact pair term and an unfavorable high packing density term, for simulation and threading," J. Mol. Biol. **256**, 623 (1996).

[6] G. L. Dignon, W. Zheng, Y. C. Kim, and J. Mittal, "Temperature-controlled liquid–liquid phase separation of disordered proteins," ACS Cent. Sci. **5**, 821 (2019).

[7] A. Opitz, "Molecular dynamics investigation of a free surface of liquid argon," Phys. Lett. A **47**, 439 (1974).

[8] C. Vega, E. Sanz, J. L. F. Abascal, and E. G. Noya, "Determination of phase diagrams via computer simulation: methodology and applications to water, electrolytes and proteins," J. Phys.: Condens. Matter **20**, 153101 (2008).

[9] G. L. Dignon, W. Zheng, and J. Mittal, "Simulation methods for liquid–liquid phase separation of disordered proteins," Curr. Opin. Chem. Eng. **23**, 92 (2019).

[10] J. R. Espinosa, A. Garaizar, C. Vega, D. Frenkel, and R. Collepardo-Guevara, "Breakdown of the law of rectilinear diameter and related surprises in the liquid-vapor coexistence in systems of patchy particles," J. Chem. Phys. **150**, 224510 (2019).

[11] S. Plimpton, "Fast parallel algorithms for short-range molecular dynamics," J. Comput. Phys. **117**, 1 (1995); P. in 't Veld, S. Plimpton, and G. Grest, "Accurate and efficient methods for modeling colloidal mixtures in an explicit solvent using molecular dynamics," Comp. Phys. Commun. **179**, 320 (2008).

[12] T. Schneider and E. Stoll, "Molecular-dynamics study of a three-dimensional one-component model for distortive phase transitions," Phys. Rev. B **17**, 1302 (1978).

[13] S. Das, Y.-H. Lin, R. M. Vernon, J. D. Forman-Kay, and H. S. Chan, "Comparative roles of charge, π, and hydrophobic interactions in sequence-dependent phase separation of intrinsically disordered proteins," Proc. Natl. Acad. Sci. U. S. A. **117**, 28795 (2020).

[14] T. S. Harmon, A. S. Holehouse, M. K. Rosen, and R. V. Pappu, "Intrinsically disordered linkers determine the interplay between phase separation and gelation in multivalent proteins," eLife **6**, e30294 (2017).

[15] D. Vitkup, D. Ringe, G. A. Petsko, and M. Karplus, "Solvent mobility and the protein 'glass' transition," Nat. Struct. Biol. **7**, 34 (2000).

[16] I. V. Volgin, S. V. Larin, E. Abad, and S. V. Lyulin, "Molecular dynamics simulations of fullerene diffusion in polymer melts," Macromolecules **50**, 2207 (2017).

[17] B. Kresse, M. Hofmann, A. F. Privalov, N. Fatkullin, F. Fujara, and E. A. Rössler, "All polymer diffusion regimes covered by combining field-cycling and field-gradient ^{1}H NMR," Macromolecules **48**, 4491 (2015).

[18] A. Einstein, "Über die von der molekularkinetischen Theorie der Wärme geforderte Bewegung von in ruhenden Flüssigkeiten suspendierten Teilchen," Ann. Phys.–Leipzig **322**, 549 (1905).

[19] Q. Li, X. Peng, Y. Li, W. Tang, J. Zhu, J. Huang, Y. Qi, and Z. Zhang, "LLPSDB: a database of proteins undergoing liquid–liquid phase separation in vitro," Nucleic Acids Res. **48**, D320 (2019).

[20] A. Bremer, M. Farag, W. M. Borcherds, I. Peran, E. W. Martin, R. V. Pappu, and T. Mittag, "Deciphering how naturally occurring sequence features impact the phase behaviors of disordered prion-like domains," bioRxiv (2021), 10.1101/2021.01.01.425046.
