# Supplementary material for: Targeted modulation of protein liquid–liquid phase separation by evolution of amino-acid sequence
Source: PLoS Comput Biol. 2021 Aug 24;17(8):e1009328. doi: 10.1371/journal.pcbi.1009328 (PMC8415608; doi:10.1371/journal.pcbi.1009328)
Supplement: Attachment — Submitted filename: coverletter-revision-plos.pdf [file pcbi.1009328.s010.pdf]

UNIVERSITY OF CAMBRIDGE
Department of Chemistry

Department of Chemistry
Lensfield Road
Cambridge
CB2 1EW
www.ch.cam.ac.uk

Prof. Damiano Piovesan & Prof. Arne Elofsson
PLOS Computational Biology

29 July 2021

Dear Professors Piovesan and Elofsson

Manuscript Resubmission

Thank you for your e-mail of 17 July concerning our manuscript entitled 'Targeted modulation of protein liquid–liquid phase separation by evolution of amino-acid sequence' (PCOMPBIOL-D-21-00779). We would like to thank the referees for their helpful and constructive comments, which have led to an improved manuscript. We are pleased to have been invited to submit a revised version, which, having addressed the referees' concerns, we are now doing.

We reply here point-by-point to the referees' recommendations and detail the changes we have made to the manuscript from our initial submission. We have also separately uploaded a copy of the revised manuscript indicating all changes made, with blue text corresponding to additions, while removed text is indicated in red.

In addition to the changes made to the manuscript as a result of the referees' comments, we have made a few minor stylistic changes so as better to comply with the journal's house style. In particular,

(i) we now use the PLOS LaTeX template rather than RevTeX,

(ii) we have rearranged the sub-panels of the figures from the previous 'one-column' style to wider, 'two-column' figures,

(iii) we now use a sans-serif typeface within the figures,

(iv) we have added figure titles to the start of each caption,

(v) we use the section titles as specified in the submission guidelines (in particular, we have reclassed the majority of 'sections' of the previous version into 'subsections' under an overarching 'Results' section, and the 'Conclusions' section is now called 'Discussion' instead),

(vi) we no longer number the sections, and hence some in-text references to section numbers have needed to change,

(vii) we have removed funding information from the acknowledgements and give this information in the submission system only, as instructed, and

(viii) we have added an explicit mention of the SI Appendix at the end of the manuscript.

We have also updated some references to pre-prints that have been fully published since our initial submission.

Reviewer 1 Comments

> *The authors presented a high-quality and original piece of work aiming to efficiently modulate the liquid-liquid phase separation of proteins by iteratively introducing changes in the protein sequence by a genetic algorithm. The method relies on coarse-graining the protein structure and a simple but effective fitness function, and is able to estimate the phase diagram of the protein. The build-up of the manuscript is logical, the language is clear, the results and methods are detailed and depicted*

sufficiently. I very much appreciated the use of 3 different protein systems, the comparison of different scoring models and the efforts for the validation. With the incorporation of the mostly minor points listed below, I highly recommend the publication of the article.

We thank the referee for their encouraging comments. We are very pleased to read they found our work of high-quality and original, and that they recommend it for publication.

Major point: 1. It would be very insightful to compare the genetic algorithm with a dummy algorithm that introduces random mutations (using the same number of rounds or iteration cycles).

We would like to thank the referee for suggesting this control. We have now performed the suggested test of using a dummy genetic algorithm on the FUS-PLD system and added a new supplementary figure S3 with the results. By setting the tournament size $N_{\text{tour}} = 1$ and using random replacement with an acceptance probability of 0.5, we retain the type of mutations our genetic algorithm introduces (including crossover events), while decoupling its evolution completely from our on-the-fly estimates of phase separating behaviour (i.e. the phase-diagram width). The results from this control simulation show that there is very little change in the fitness function (which is our proxy for the critical solution temperature) of FUS across this dummy evolution; by contrast, the original genetic algorithm produces a notable increase in the fitness function. Together with the fact that the critical temperature can also be purposely reduced if the fitness function is inverted (Fig. S4), adding this control allows us to strengthen the argument that the origin of the observed evolutionary driving forces indeed lies within the fitness function and is not solely a consequence of random mutations.

Minor points: 1. Fig 2B, Fig 4B, Fig 5B seem to use different colours from the colour scales, as white colour does not appear on the blue-brown-orange and blue-purple-red colour scales, only on the figures. Please show the appropriate colour scales.

Thank you for pointing this out. It seems the colour bar as we had previously implemented it did not render correctly in certain PDF viewers (in particular the PDF viewer embedded in Firefox, and perhaps others based on the same engine). The middle colour in the gradient was absent in such viewers, but showed as intended in other viewers (such as Okular and Adobe Reader). It is apparently a known bug in the Firefox PDF viewer that this happens. As a workaround, we have now produced all colour bars using a different method, and the colours now display correctly in all the PDF viewers we have tried. Since figures need to be submitted to PLOS Comput. Biol. in a different format (encapsulated PostScript, rather than a PDF), we will check the proofs produced very carefully with different viewers to ensure this is not a problem with the final version of the paper.

2. Statistical significance is unclear in the following sentence: "This pair correlation function exhibits a more pronounced nearest-neighbour maximum, indicating a greater degree of local structure and an increase in the number of nearest-neighbour beads compared to the WT..." Could you please add the appropriate statistics?

We have now computed the pair correlation function using a finer grid (Fig. 2(C)), which allows the maximum to be better characterised, and in turn makes a quantitative comparison between the two sets of simulations, now explicitly given in the manuscript, more meaningful. Error bars obtained from the standard deviations of averages computed for independent data runs are of the order of the line thickness of the figure; we now explicitly comment on their magnitude in the manuscript, as requested.

3. In Fig 3A the colour scale of hydrophobicity should match with that of Fig 2B [0,1] in order to avoid interpretation bias.

In order to remove the scope for interpretation bias, we now use a different colour scheme in Fig. 3A that cannot be confused with that of Fig. 2B. We now explicitly plot the *difference* between the mean hydrophobicity parameter of the amino acids in the chunk relative to the hydrophobicity parameter of glycine. In the original version, this difference was implicit in the colour only, but we agree with the referee that this could easily have been misinterpreted as corresponding to the same colour scheme as used in Fig. 2B.

4. Please add the correlation coefficient to the following sentences of the main text: "Fig. 3(A) shows the results of a glycine scan in sequential chunks of 6 amino acids, projected onto the chunk-averaged hydrophobicities of the WT protein. The curves anti-correlate for most of the sequence..." & "Our comparison indicates that there is a good positive correlation between the predictions of the sequence-dependent coarse-grained models we have used and the experimental results..."

We have now added a correlation coefficient for the anti-correlation of the results in what was Fig. 3(A) [now Fig. 3(B)] to the text. We had previously reported the correlation coefficients for the experimental validation in the supplementary information, but now report it in the main text [Fig. 7], as advised.

5. When simulating two-component systems, the RNA:protein stoichiometry was chosen to be 1:7, which I believe is not physiologically relevant. What about choosing a more relevant stoichiometry for an additional GA run?

Thank you for this suggestion. Our intention when testing the behaviour of our genetic algorithm for the IDRs of FUS and hnRNAP1 in the presence and absence of RNA is only to demonstrate that our method is sensitive enough to evolve the amino-acid sequence of a given protein intentionally to favour (or disfavour, as the case may be) its phase-separating behaviour while taking into account the condensate composition. Using a different RNA:protein ratio would undoubtedly change the final evolved protein sequences. However, our work aims to present a new computational framework that can be used to optimise the design of amino-acid protein sequences with desired phase behaviour; we focus on IDRs rather than on physiological proteins/systems. While in the future, investigating evolution of the amino-acid sequences that promote protein phase behaviour at different RNA concentrations would indeed be very interesting, this will only become possible at physiological RNA:protein ratios when the challenge of determining what the physiological compositions of condensates are is achieved by the community. We have now clarified this in the manuscript, and added a few references that discuss this point further.

6. Please add the correlation coefficient for the simulated data using KH and cation-pi models. This would quantify the disagreement between the two models.

We have now added the model–model Pearson correlation coefficient in the 'Experimental validation' section.

7.i. The word amino acid is written as 'amino acids' or 'amino-acids' inconsistently. Please remove/add the hyphens. 7.ii. The same issue was found for 'genetic algorithm' vs 'genetic-algorithm'.

Having corrected a couple of slip-ups on this front, we believe we are now using hyphenation in a fully consistent way: whenever each phrase acts as a compound modifier, it is hyphenated, whilst it is not when it is a nominal phrase. So, for example, we would write 'an amino-acid sequence' and 'a sequence of amino acids'. We have not found any information about the PLOS house style for compound adjectives, but this approach to compound adjectives is, we feel, reasonably standard.

8. I'm very happy to see that the data will be made publicly available. However, it is a pity it is unavailable under the review. Please make sure that the analysis scripts to reproduce the evaluation of the simulations results are also archived with the simulation files.

We originally intended to submit all the raw data to our University's data repository. However, changing any files, for example after peer review, would result in a new DOI, and this is why we intended to upload the final archive upon acceptance, once it is clear precisely what data need to be included. However, it seems to be possible to include such supporting data directly in PLOS Comput. Biol., rather than having to rely on a separate data repository, and so we have now included in the online submission interface all the supporting data underlying the study. This archive includes the raw data used in all the figures, in both the manuscript and the SI; a series of LAMMPS scripts that can be used to generate the simulation data; analysis scripts used to post-process the data; and a full listing of all sequences across all GA runs reported alongside their phase-diagram widths (which are used in the fitness function).

Reviewer 2 Comments

The manuscript by Lichtinger and colleagues presents an interesting computational approach for investigating the effect of sequence modifications in Intrinsically Disordered Regions (IDRs) in three well-studied proteins (FUS, hnRNPA1, LAF1) known to undergo homotypic liquid-liquid phase separation (LLPS) and their LLPS potential. In particular, they propose a simple to compute fitness function to guide a Genetic Algorithm (GA) coupled with coarse-grained molecular dynamics (MD) simulations towards evolving sequences that have desired (increased or decreased) LLPS tendency compared to the wild type.

The work presented is novel, and aims to tackle an important and timely problem. Overall, the manuscript is well written, starting with a brief -yet informative- introduction on the current knowledge in the current knowledge of the driving forces behind LLPS. Then the authors describe a computationally tractable approach to tackle the problem of training a GA to shift the phase behavior of a starting (WT) sequence to a local optimum. This becomes feasible by introducing the difference in composition densities between the protein-rich and protein-poor phases in an appropriately designed coarse-grained MD simulation of the protein region in question. The authors present results on three IDRs know to drive LLPS in vitro and in vivo, and validate their results against more detailed, all-atom MD simulations. The results presented herein do not identify a single driving force for LLPS but provides some useful insights on parameters other than charge, hydrophobicity and their patterning along sequences that should also be considered when rationally designing mutations for promoting/inhibiting LLPS. In addition, examples where RNAs are introduced in the FUS/hnRNPA1 simulations are presented, aiming to demonstrate the potential of the proposed approach to handle more complex systems.

We thank the reviewer for their encouraging comments. We are especially pleased to hear that they found that our work is 'novel, and aims to tackle an important and timely problem'.

Below are some points that I believe warrant some attention by the authors. Page numbers refer to the page number of the final PDF file produced in the Editorial Manager system and not the page numbers inserted by the word processing tool.

+ Specific comments- The manuscript is accompanied by detailed supplementary material. Even though some methodological details (especially those referring to existing methods) are safe to reside in the supplement, I would prefer a large portion of this material to be transferred to the main text (e.g. S4-S9, S12).

Thank you for this suggestion. We have now moved most of the sections above to the main text, as the referee suggested. In particular, the former Fig. S4 (the radial distribution function) is now shown as Fig. 2(C); the former Fig. S6 (hnRNPA1 results) is now incorporated into Fig. 4; the former Fig. S7 (LAF1 results) is now incorporated into Fig. 5; the former Section S12 (except for the enumeration of sequences) has been merged into the 'Experimental validation' section of the main text, including Fig. 7.

The former Fig. S5 (chunk shuffling runs) is merely an intermediate stepping stone in obtaining the results already shown in Fig. 3 of the main text, and as the final results are already shown, we feel it is appropriate to keep the extended figure in the SI.

The former Figs S8 (cation–π model) and S9 (liquid character of phases) are essentially benchmarks for our results. The former confirms that the results are not overly sensitive to the choice of model, while the latter is just a sanity check to make sure that the system remains liquid-like under all conditions we have considered, but beyond this, we do not analyse it further. We would prefer to keep these in the SI, as they do not fit the main narrative of the manuscript.

- Regarding the results presented in Fig 2A, it is apparent that when FUS-WT is evolved to either high or low Tc, Ser residues seem to decrease (same holds for Gln and Thr even though these changes seem weaker). Could this observation be explained by some positional preferences? Please discuss. In

addition, in Fig 2B it is mentioned that "Trends in hydrophobicity and σ are largely correlated". It is unclear though if this correlation is somehow quantified. In addition, there exist several clusters of successive residues that seem to not have undergone mutation: doe these correspond to cases where mutations in these positions were not explored by the GA or that such mutants were rejected during the simulation? Are there any particular features of the WT sequences in these stretches? Please discuss.

We believe the frequency of serines decreases in both cases primarily on statistical grounds: it it very common in the original sequence, and so many attempted mutations will entail this residue. Some of these will lead to a significantly increased fitness and are therefore taken forward by the genetic algorithm. Others, by contrast, may not make much difference, since several amino acids have a similar set of interaction parameters in this model. There can therefore be a stochastic change towards an equal distribution of such largely 'equivalent' residues, even when serines may have been favoured on other (biological) grounds. We previously made a note to this effect in footnote 79; however, PLOS house style is to include footnotes as part of the references, and this led to its becoming buried deep within the long bibliography. We have now moved this footnote into the main text, thus making it much more prominent.

Regarding the correlation between hydrophobicity and σ, we have now quantified the statement with a Pearson coefficient in the text. We already explicitly discussed that there are outliers in the trend, so the correlation coefficient shows a moderate correlation (of ~0.4).

Finally, the referee asks an important question regarding whether the stretches that remained largely unchanged during the course of the evolution did so because changes were not attempted to begin with, or not accepted by the GA. In the case of FUS and hnRNPA1, we have now clarified that essentially all residues had mutations attempted on them; they remain unchanged because such mutations were rejected by the algorithm as less fit. For the case of LAF1, which entails a shorter set of simulations because of its larger size, we now explicitly highlight in Fig. 5(D) those residues where no mutations were attempted in the course of these runs.

- Regarding the Glycine-scans presented (pg. 12), a chunk size of 6 AAs was chosen. However, the results shown in Fig 3B are interpreted by the authors that segments of 2-3 successive AAs are crucial in driving LLPS in FUS. Would it be more meaningful if the Glycine-scans were performed with a chunk size of 2 or 3 AAs? Another question related to Fig 3B: when chunks a shuffled it is not necessary that all residue types are changed - is this taken into account?

We appreciate the referee's question, as it led us to realise that the order in which we presented the glycine scans and chunk shuffling results was perhaps the wrong way round. The glycine scans allow us to investigate the larger-scale behaviour: indeed choosing a chunk size of 6, which is larger than the characteristic length scale of 2–3, ensures that we are not disrupting the underlying stickers and spacers, allowing us to focus on the overall effect of hydrophobicity. Although using a smaller chunk size in glycine scans may be appealing, the chunks of 2 or 3 consecutive amino acids that form 'stickers' do not necessarily correspond to blocks obtained by an even spacing from the start to the end of the sequence, and so in our opinion we would be unlikely to gain further insight by making the chunks smaller. To explain our reasoning more clearly in the manuscript, we have swapped the order in which we present chunk shuffling and glycine scans, and explain that we have chosen a chunk size of 6 in glycine scans because it is larger than the characteristic hydrophobic patterning length scale of 2–3 amino acids.

We also now explain explicitly that when shuffling chunks, it may indeed be the case that some residues that are 'swapped' may be of the same type.

- It is unclear what the two evolved variants of LAF1 mentioned in pg. 16 refers to. Please explain how these variants were selected and what is their relevance.

The two LAF1-derived sequences chosen are merely representative examples of sequences with similarly high fitness but very different energetics. We have now clarified this in the manuscript.

- Under section (V) the authors interpret the results shown in Fig 6A in a descriptive way. They mention wrt FUS that "the creation of more lysine (K) residues is largely offset by creating fewer arginine (R)

residues, which have the same charge". However, simple inspection of Fig 6A indicates that net positive charge (especially if His is also taken into account) seems to clearly increase in the presence of RNA (while negative charge decreases). With minor only changes involving negatively charged residues I suspect that there might be some misinterpretation here (or I have missed some crucial detail).

Indeed, the overall charge increases in Fig. 6(A) both with and without RNA. As we show in Fig. 6(B)(i), there is a very slightly larger positive charge with RNA than without, although the error bars are significant. The statement about K vs R residues we have made was meant to emphasise that although there is a significant difference in the frequencies of these amino acids in the two cases (with and without RNA), the overall charge is not drastically different, but is in both cases larger than the WT sequence. We were attempting to contrast this behaviour to that of hnRNPA1, where the difference in charge for the cases with and without RNA is very large by comparison. We have now rephrased the offending sentence to make it clearer what we are trying to compare and contrast.

+ Minor comments and typos- pg. 8. When mentioning "sufficient number of copies": How is this number selected?

The number of copies of the protein in the simulation must be sufficiently high that finite-size effects do not dominate the system's bulk behaviour. This means that at temperatures well below the critical point, the liquid-like phase must form a sufficiently large proportion of the overall system that it is possible to determine its average density. We have checked that we are safely in this regime by explicitly probing finite-size effects, namely by reducing the system size by ~30 % and verifying that the phase behaviour and the computed properties of the phases are not affected by this change. We have not simulated 'minimal' systems, but have kept a reasonable margin to ensure that changes in sequence can not easily lead to prominent finite-size effects.

We have now added a note summarising our finite-size effect verification to the supplementary information document.

- pg. 9. "[(2)]" should better read "[(Equation 2)]. The same also holds for the legend of Fig 1 (B).

Thank you for this; we have now explicitly added 'Eq.' whenever we refer to an equation.

- pg. 16. It is unclear to me (a) why the GA progression is slower with this system and (b) why are finite-size effects are more pronounced? The LAF1 IDR is approx the same length compared to the respective FUS domain, so I suspect it is not a matter of length. Could you please elaborate?

We have now clarified that what we are referring to is that the simulations are slower, rather than the efficacy of the genetic algorithm itself. The latter is also true to an extent, but it is potentially ambiguous:. The shape of the phase diagram means that the fitness function in the case of LAF1 is less well correlated with the critical temperature than in previous cases (e.g. for hnRNPA1, a fitness function increase of 20 % corresponds to an increase in the critical temperature of 50 %, whilst for LAF1 an increase in the fitness function of 30 % only increases the critical temperature by 25 %). However, the fitness function itself increases faster for LAF1 than for hnRNPA1 as a function of the round.

The simulations are so much slower because of the larger system sizes required and the high charge content (22 % of all residues are charged), resulting in more long-ranged interactions that are slower to compute. We can speculate why finite-size effects are more significant, but we have not analysed this in sufficient detail to warrant inclusion in the manuscript. We suggest that the E and R residues at the start and end of LAF1, which have the same charge, repel each other more in the interfacial region, which dangling ends should occupy preferentially for entropic reasons. This means that the interfacial region is extended and the corresponding 'bulk' region smaller, and hence we need a larger system to characterise the bulk phases. While hnRNPA1 also has some charged residues relatively early on, the overall charge content is lower, and since the polymer is shorter, the danging ends are correspondingly less likely to be in the interfacial region due to the lower entropic driving force.

- pg. 16. "... the amino acids early in the sequence ...", does it refer to the most N-terminal residues? Please clarify.

Yes, this is precisely what we meant. We have clarified this in the text to make it unambiguous.

> *- pg. 19. Since the preprint by Bremer et al [ref. 23] is quite new and most readers may not be very familiar with the naming convention of hnRNPA1 analogues (+7R and +7F–7Y), please consider briefly describing them in the text or supplement for clarity.*

We have now added an explanation of the naming convention, and we have highlighted the residues that change relative to the wild type in the SI.

> *- pg. 21. "encourage or inhibit" might better read as "promote or inhibit".*

We have made this change; thank you for the suggestion.

> *- pg. 22. The symbol P refers to denote either the mutation rate (point 1) or the set of parents (point 2) which could be misleading.*

Thank you for spotting this. We have now removed this clash of symbols by referring to the mutation rate only by name and not using any symbol.

> *- pg. 31. Under S1, in the mutant hnRNPA1 sequences it could be useful to highlight mutations wrt the WT.*

We are unsure what precisely the referee had in mind here. As far as we can tell, p. 31 of our original submission starts with 'S3 Reducing the critical temperature', which is not related to hnRNPA1, while S1 is the section on computational details. Perhaps S10 was meant, which listed the sequences of the original proteins, or S12, which listed the sequences of the experimentally studied variants of hnRNPA1. In any case, we have now highlighted the changes in sequence of the hnRNPA1 variants, both from evolutionary runs and the experimental ones used for validation, relative to the wild type, and we hope this is what the referee was suggesting we should do.

Reviewer 3 Comments

> *The paper entitled "Targeted modulation of protein liquid-liquid phase separation by evolution of amino-acid sequence" by Lichtinger et al. addresses an important and challenge topic in the field of LLPS, aiming to understand how single point amino acid variations affect the driving forces in LLPS, modulating it. They followed a novel computational methodology by combining genetic algorithms and sequence-dependent coarse-grained protein models, to evolve amino-acid sequences of phase separating IDPs. The authors performed a deep analysis with a very precise and rigorous methodology on the different sequence properties as hydrophobicity and residue charge in the wild type and the evolved sequences of PLD of FUS, hnRNA and LAF1, well-studied proteins in the LLPS field. Their observations suggest that not all proteins that phase separate are governed by the same the driving forces. Moreover, authors validate their findings seen by fitting the predictions against experimental phase diagrams for a set of sequences of hnRNA1, and by using a more realistic and accurate ABSINTH model.*
>
> *Authors present a novel and efficient way to evolve naturally occurring phase-separating protein sequences, however my only concern regards to generalization of this method to other proteins and its robustness. Since only few protein sequences have been used in this work, I suggest extending the discussion about how this method could be applied at large-scale on the current LLPS databases (e.g., PhasePro, PhaSepDB, etc). This may allow to identify group of proteins that exhibit LLPS and are governed by similar driving forces. After addressing this minor comment, I recommend this work to be published in PLOS Computational Biology.*

We thank the reviewer for their encouraging comments, and recommending publication of our work after revision. This is a very good point; the databases provide a very useful set of starting points of known phase-separating proteins. By applying the method proposed in our manuscript systematically to a wide range of different proteins,

we may be able to determine classes of proteins whose LLPS is governed by similar driving forces, providing new insight, a possible means of classification, and perhaps even suggest in due course which systems may have been underexplored experimentally. We have added a comment to this effect to the conclusion of the manuscript.

Yours sincerely

Simon Lichtinger
Adiran Garaizar
Rosana Collepardo-Guevara
Aleks Reinhardt
